# Supplementary material for: Psychometric evaluation of the Persian nursing students’ learning self-efficacy instrument
Source: PLoS One. 2025 Sep 2;20(9):e0331435. doi: 10.1371/journal.pone.0331435 (PMC12404491; doi:10.1371/journal.pone.0331435)
Supplement: S3 File — (DOCX) [file pone.0331435.s001.docx]

# Supporting file 3. Standardized Factor Loadings for the CFA Model of the Persian NLSE Instrument (n = 240)

| Item | Construct | Factor Loading |
| --- | --- | --- |
| 1 | Conceptual Understanding | 0.78 |
| 2 | Conceptual Understanding | 0.85 |
| 3 | Conceptual Understanding | 0.81 |
| 4 | Higher-order Cognitive Skills | 0.69 |
| 5 | Higher-order Cognitive Skills | 0.73 |
| 6 | Higher-order Cognitive Skills | 0.75 |
| 7 | Higher-order Cognitive Skills | 0.79 |
| 8 | Higher-order Cognitive Skills | 0.81 |
| 9 | Practical Work | 0.71 |
| 10 | Practical Work | 0.74 |
| 11 | Practical Work | 0.76 |
| 12 | Practical Work | 0.8 |
| 13 | Practical Work | 0.77 |
| 14 | Practical Work | 0.82 |
| 15 | Everyday Application | 0.76 |
| 16 | Everyday Application | 0.74 |
| 17 | Everyday Application | 0.79 |
| 18 | Nursing Communication | 0.72 |
| 19 | Nursing Communication | 0.75 |
| 20 | Nursing Communication | 0.78 |
| 21 | Nursing Communication | 0.8 |

Note: All factor loadings were statistically significant at p < 0.001.
