# Supplementary material for: Psychometric evaluation of the Persian nursing students’ learning self-efficacy instrument
Source: PLoS One. 2025 Sep 2;20(9):e0331435. doi: 10.1371/journal.pone.0331435 (PMC12404491; doi:10.1371/journal.pone.0331435)
Supplement: Attachment — Submitted filename: Response to Reviewers.docx [file pone.0331435.s003.docx]

**Response to the Editor and Reviewers**

We sincerely thank the Academic Editor and the reviewers for their constructive and insightful comments, which have been invaluable in improving the quality of our manuscript. Each point raised has been carefully considered and addressed in detail. All revisions are clearly marked using Track Changes within the manuscript. In summary, we have revised the manuscript in accordance with the suggestions and recommendations provided. We are grateful for the time and attention devoted to our work during this revision process.

Best regards,

Corresponding author

| **Author’s responses** | | **Comments of Editor** |
| --- | --- | --- |
| Thank you for your valuable comments and the opportunity to revise our manuscript.  As requested, we are submitting two versions of the revised manuscript: 1. A marked-up version with track changes labeled “Revised Manuscript with Track Changes”, highlighting all modifications made. 2. A clean version without tracked changes labeled “Manuscript”. | | A marked-up copy of your manuscript that highlights changes made to the original version. You should upload this as a separate file labeled 'Revised Manuscript with Track Changes'.  An unmarked version of your revised paper without tracked changes. You should upload this as a separate file labeled 'Manuscript'. |
| Thank you very much for your thoughtful comments and the opportunity to revise our manuscript. In response to your guidance, we have thoroughly revised the manuscript and carefully ensured that it adheres to PLOS ONE’s style requirements, including the appropriate file naming conventions. | | 1. Please ensure that your manuscript meets PLOS ONE's style requirements, including those for file naming. The PLOS ONE style templates can be found at |
| Thank you very much for your careful review and valuable comment. We apologize for the error caused by a copying mistake. The correct IRB approval number (IR.ARUMS.REC.1403.174) has now been updated and corrected in the manuscript. | | 2. We have noted that the IRB document (IR.ARUMS.REC.1403.174) you have supplied does not match the approval number stated in your manuscript (IR.ARUMS.REC.1403.17). Please update the correct IRB number (IR.ARUMS.REC.1403.174) in the manuscript. |
| **Author’s responses** | | **Comments of Reviewer 1** |
| **Introduction** | | |
| Thank you for your valuable comment. In response, we have revised the introduction to include a clear explanation of Bandura's social cognitive theory and its relevance to learning self-efficacy. | 1- Given that your research topic is in the field of self-efficacy, it is better to refer to Bandura's self-efficacy theory and write about the relationship of this theory to learning self-efficacy. | |
| Thank you for raising this important point. To the best of our knowledge and based on a thorough review of the literature, the Nursing Students’ Learning Self-Efficacy Scale (NILES), developed by Yin et al. (2024), has not yet been psychometrically evaluated outside of Taiwan. Therefore, our study represents the first effort to translate and to assess psychometrically the NILES instrument in a non-Taiwanese context, specifically among Persian-speaking nursing students in Iran. | 2- Have psychometric studies of the NILES instrument been conducted in other countries? Please list the psychometric results in other countries. | |
| Thank you for your insightful comment. In response, we have revised the Introduction section to include a summary of the study that developed and validated a tool for assessing Iranian nursing students’ clinical performance self-efficacy. | 3- In Iran, a self-efficacy tool for clinical performance of nursing students has been developed and psychometrically tested. It is better to mention in the introduction the results of this study and how this tool differs from the tool you are studying. Did you only examine the students' theoretical learning self-efficacy in this study or was their clinical learning self-efficacy also examined? | |
| Thank you for your suggestion!  In the end, it was added to the introduction. | 4- It would be better to add this at the end of the introduction: "The psychometric properties of this tool, in addition to improving educational processes, can help predict and evaluate the success of students in completing coursework and academic progress." | |
| Thank you very much for your valuable suggestion. The sentence at the end of the introduction has been edited as requested to read "…multidimensional learning self-efficacy tool…". | 5- At the end of the introduction, edit the sentence, instead of "... …multidimensional self-efficacy learning tool" write "…multidimensional learning self-efficacy tool..." | |
| **Methods** | | |
| Thank you very much for your valuable feedback. The type of study has been added in the study design section as per your suggestion. | | 6- What was the type of study? |
| Thank you very much for your valuable comments. Exploratory factor analysis was conducted after face and content validity assessments, and the related information has been added in the first section on construct validity to reflect the proper order and sequence of the validity methods used in this study. | | 7- You also used exploratory factor analysis in this study. This validity is usually done after face and content validity. Please write the method in order and sequence. |
| Thank you very much for your valuable comments. Information regarding the sample size for exploratory factor analysis (EFA), its determination method, the minimum acceptable factor loading, and the indicators used, along with their acceptable values, has been added in the first section of the construct validity in the Methods part of the manuscript with appropriate reference. | | 8- What was the sample size for exploratory factor analysis and how did you determine it? What was the minimum acceptable factor loading for exploratory factor analysis? What indicators were used to conduct the analysis and what are their acceptable values? Reference should be given. |
| Thank you for your insightful comments and suggestions. References have been provided as requested. | | 9- Please write the acceptable values of the fit indices used (CFI, RMSEA, PNFI, TLI, PCFI) in the CFA to confirm the model? References should be provided. |
| Thank you for your valuable feedback. In response to your suggestions, I have provided the acceptable values for Cronbach's alpha, which measures internal consistency, along with appropriate references. | | 10-The acceptable Cronbach's alpha values for internal consistency should be written. |
| Thank you very much for your valuable comments. In response to your inquiry, we have reported the Intraclass Correlation Coefficient (ICC) for the test-retest reliability to assess the stability of the instrument over time. Additionally, acceptable values for both Cronbach’s alpha and omega have been added to the manuscript. As noted, omega values above 0.70 are generally considered acceptable, indicating good internal consistency, similar to Cronbach’s alpha.. | | 11- Have test-retest and omega been used for the reliability of the instrument, or has the ICC of the instrument also been examined? What are the acceptable values for the omega test? |
| **Results** | | |
| Thank you very much for your valuable comments. The exploratory factor analysis (EFA) results have now been included in a table, similar to the presentation of the CFA results in Table 2, and this table has been added to the Results section of the manuscript. | | 12- It would be better if the EFA results were also shown in the table, like the CFA results shown in Table 2, because in this study, you mentioned EFA in the method section but did not provide any data or information in the results section. |
| Thank you very much for your valuable comment. The omega reliability coefficient results have now been included and reported in Table. | | 13- Why are there no omega reliability coefficient results in Table 4? |
| **Discussion** | | |
| We sincerely thank the reviewer for their valuable feedback, which has significantly improved the manuscript. This comment has been systematically addressed. | | 14- In the discussion section, only one study (reference 17) has been used, which is the study of the original designer of the NILSE questionnaire. I think you should use similar studies in the field of instrument psychometrics and close to your study in the discussion section. |
| **Comments of Reviewer 2** | | **Comments of Reviewer 2** |
| **Title** | | |
| Thank you for your valuable comment. It was fixed. | | Comment: The title is descriptive but slightly long. Consider simplifying it to improve readability (e.g., "Psychometric Evaluation of the Persian Nursing Learning Self-Efficacy Instrument"). |
| **Abstract** | | |
| Thank you for your valuable comment. It was fixed. | | Comment 1: Consider adding a stronger concluding sentence to emphasize the implications or practical value of the instrument. |
| Thank you for your valuable comment. It was fixed. | | Comment 2: Avoid using abbreviations like PCFI or PNFI in the abstract without defining them. |
| **Introduction** | | |
| Thank you for your valuable feedback. We appreciate your suggestion regarding the introduction. We have carefully reviewed the section and revised it to eliminate repetitive points about self-efficacy and academic performance. The narrative is now more concise while maintaining a comprehensive background to clearly establish the importance of the study. | | Comment 1: The introduction provides a comprehensive background, but the narrative could be more concise. Try to eliminate repetitive points about self-efficacy and academic performance. |
| Thank you for your constructive suggestion. In response, we have added a clearly marked paragraph at the end of the introduction under the subheading “Objective,” which explicitly states the research aims. | | Comment 2: Consider ending the introduction with a clearly marked paragraph stating the research aim(s) or hypotheses under a subheading such as “Objective”. |
| **Methods** | | |
| Thank you for your valuable feedback. We have conducted Exploratory Factor Analysis (EFA) and included the results in the revised manuscript. These results have provided insights into the factor structure and allowed us to better understand the cultural-linguistic adaptations of the instrument. We will present the EFA results and their implications for Confirmatory Factor Analysis (CFA) in the updated version of the paper. | | **Study Design and Setting**  Comment: The study is described as a methodological cross-sectional design, which is appropriate for psychometric evaluation. However, the absence of exploratory factor analysis (EFA) as a first step is notable. Even if CFA is justified due to prior structure (from Yiin et al., 2024), a rationale for not including EFA should be explicitly stated. This is e specially important given the cultural-linguistic adaptation, which may impact factor structure. |
| Thank you very much for your valuable comment. A detailed explanation regarding the use of convenience sampling and its potential impact on the generalizability of the findings has been added to the Methods section of the manuscript. | | **Sampling and Participants**  Comment 1: The authors report using a convenience sampling method, which is common in validation studies, but carries inherent selection bias. This limitation is acknowledged briefly in the discussion, but should also be addressed in the methods section with more detail about how it might affect generalizability. |
| Thank you very much for your valuable comment. In the Methods section, we have added a detailed explanation of how the 250 students were approached and recruited, as well as how non-responses and dropouts were handled, to clarify the sampling process and its implications for the study. | | Comment 2: The sampling size rationale (10:1 item-to-sample ratio) is standard and supported by citation. However, the final sample (n=240) exceeds the calculated requirement (n=210), which is positive. Still, there is no description of how the 250 students were approached or recruited, nor how non-responses or dropouts were handled. |
| Thank you very much for your valuable comment. In the Methods section, we have clarified the inclusion and exclusion criteria by specifying that formal informed consent was obtained from all participants. Additionally, we explained that incomplete questionnaire submissions—defined as those with missing responses in key sections—were excluded entirely from the analysis. The number of such incomplete responses and how they were handled have also been detailed to enhance transparency. | | Comment 3: Inclusion and exclusion criteria are mentioned, but they are vague. For example, the phrase "willing to participate" should be supported by more formal consent processes, and "incomplete questionnaire submissions" needs further clarification—were partial completions excluded entirely? How many? |
| **Translation Process** | | |
| Thank you very much for your valuable comment. According to standard psychometric procedures, face validity is considered the first step of validation after translation. In this study, face validity was assessed qualitatively through cognitive interviews to ensure the clarity and cultural appropriateness of the translated items before proceeding to further psychometric evaluations. Therefore, although a separate pilot study was not conducted, this qualitative phase served as an initial evaluation of the instrument’s validity**.** | | Comment: The process followed WHO translation guidelines, which is appropriate and strengthens methodological rigor. The description is comprehensive and demonstrates cultural and linguistic sensitivity. It would be helpful to specify if any pilot testing or cognitive debriefing was conducted with the translated items before formal psychometric evaluation. |
| **Validity Assessments** | | |
| Thank you for your insightful comment regarding the face validity assessment. We appreciate your feedback, and I want to assure you that the integration of feedback from students and the expert panel has been implemented. Specific revisions were made to clarify this process, ensuring that it is well-documented and transparent. | | Face Validity:  Comment: The face validity assessment includes both qualitative (interviews) and quantitative (impact score) methods, which is excellent. However, the process for integrating feedback from students and the expert panel is not fully detailed. What kind of revisions were made? |
| Thank you for your valuable comment regarding the content validity assessment. We appreciate your suggestion, and I want to confirm that we have implemented the change to report CVR and CVI values item-by-item in supplementary tables, rather than just providing a range. | | Content Validity:  Comment: CVR and CVI assessments were well-executed, using Lawshe’s method and relevance rating scales. However, authors should report CVR/CVI values item-by-item in supplementary tables, not just as a range (e.g., “0.8 to 1”) |
| Thank you very much for your valuable comment. Exploratory factor analysis (EFA) was conducted in this study, and the related information has been added in both the Methods and Results sections to provide data-driven insights alongside the confirmatory factor analysis (CFA). | | Construct Validity:  Comment: The use of CFA alone is limiting. As mentioned, EFA could have offered data-driven insights into whether the original structure is fully retained in the Persian context. |
| We sincerely thank the reviewer for their constructive feedback. In response to the suggestion to enhance transparency of the CFA results, we have taken the following steps: (1) included a path diagram as Figure 1 in the Results section showing standardized factor loadings, latent variable correlations, and error terms; (2) provided detailed factor loadings for all items across the five constructs in Supplementary Table 1, with all values exceeding 0.3 (p < 0.001); and (3) made the complete table available as supplementary material. These additions offer both visual and numerical representations of the factor structure to improve clarity and reproducibility of our findings. | | CFA indices are well reported (χ²/df, RMSEA, CFI, etc.) and acceptable, but a visual representation of the factor loadings (e.g., a table or path diagram) would improve transparency. |
| Thank you very much for your valuable comment. Although the sample size for the test-retest reliability phase was relatively small (n=30), this number was chosen based on practical considerations and is consistent with similar psychometric studies. We acknowledge that a larger sample size could provide a more precise estimation of stability. Therefore, this limitation has been addressed in the manuscript, and we recommend that future studies include a larger sample for test-retest analysis to enhance the robustness of reliability assessment. | | Reliability Testing  Comment: The study used Cronbach’s Alpha, Omega coefficient, and test-retest (ICC), which provides a robust assessment of reliability. Yet, the sample size for test-retest (n=30) is relatively small and could affect stability estimation. Justify this choice or consider discussing it as a limitation. |
| We sincerely appreciate the reviewer's valuable feedback regarding convergent and discriminant validity assessment. As suggested, we have now included the relevant formulas for Composite Reliability (CR) and Average Variance Extracted (AVE) in the methodology section, along with appropriate references to support calculation procedures. This addition enhances the transparency and replicability of our measurement validation approach. | | Convergent and Discriminant Validity  Comment: Composite Reliability (CR), Average Variance Extracted (AVE), and Maximum Shared Squared Variance (MSV) are reported and interpreted correctly. It is a strength that these indices are used instead of relying solely on traditional correlation methods. However, formulas or calculation details for these measures could be briefly included or referenced to support replicability. |
| Thank you very much for your valuable and constructive comments. We have carefully addressed your suggestions by providing more detailed information on the structural equation modeling (SEM) parameters, including model fit indices, modification indices, and standardized residuals in the Data Analysis section. Additionally, confidence intervals for the ICC, factor loadings, and fit indices have been included to enhance the rigor and interpretability of the results. These revisions have been incorporated accordingly. | | Statistical Analysis  Comment 1: The software used (SPSS and AMOS v.24) is standard. The description of statistical procedures is mostly accurate, but overly general. The structural equation modeling (SEM) is mentioned, yet no model parameters, modification indices, or residuals are discussed. |
|  |  | Comment 2: The study uses p < 0.05 as a threshold for significance, but does not report confidence intervals for ICC, factor loadings, or fit indices—these should be added for more rigorous interpretation. |
| **Results** | | |
| Thank you very much for your careful and valuable comment. We have reviewed the tables and ensured that all numerical values are now presented with a precision of two decimal places where appropriate. | | Comment 1: Some tables (e.g., descriptive stats) include excessive decimal places. Limit precision to two decimal places where appropriate. |
| Thank you very much for your valuable comment. A brief narrative description of Figure 1 has been added to enhance reader's interpretation of the CFA model, explaining the latent constructs, observed variables, and the relationships depicted in the path diagram. This addition aims to clarify the measurement structure and improve the overall understanding of the CFA results. | | Comment 2: Add a brief narrative description of Figure 1 to enhance reader interpretation of the CFA model. |
| **Discussion** | | |
| We sincerely thank the reviewer for their valuable feedback, which has significantly improved the manuscript. All comments have been systematically addressed. | | Comment 1: The discussion summarizes findings appropriately but could benefit from more critical reflection. For instance, why might certain subscales have lower reliability? |
|  |  | Comment 2: Compare your findings more directly with the original NLSE validation study by Yiin et al., particularly regarding cultural adaptation differences. |
|  |  | Comment 3: Consider restructuring the discussion to follow the order of your findings (face validity, content validity, construct validity, reliability) for better coherence. |
| **Conclusion** | | |
| We sincerely thank you for your valuable feedback and careful consideration. Your insightful suggestions significantly improved the conclusion of our paper by incorporating practical applications (e.g., utilizing the NLSE for curriculum planning, educational resource allocation, and student support services) and highlighting future research directions (e.g., multi-center studies, longitudinal analyses). These revisions have strengthened the alignment of our findings with real-world educational practices and expanded the potential utility of the NLSE instrument. Thank you for your invaluable contribution to advancing the quality and relevance of this research. | | Comment: The conclusion is aligned with the study’s findings. You may strengthen it by briefly suggesting practical applications (e.g., curriculum planning, student support services) and future research directions. |
